# Association of *STAT4* and *PTPN22* polymorphisms and their interactions with type-1 autoimmune hepatitis susceptibility in Chinese Han children

**DOI:** 10.18632/oncotarget.17458

**Published:** 2017-04-27

**Authors:** Xiaofeng Li, Huiqin Chen, Yun Cai, Pingping Zhang, Zhuanggui Chen

**Affiliations:** ^1^ Department of Pediatrics, The Third Affiliated Hospital of Sun Yat-sen University, Guangzhou 510630, China

**Keywords:** autoimmune hepatitis, STAT4, PTPN22, single nucleotide polymorphism, interaction

## Abstract

**Aims:**

To investigate the impact of *signal transducer and activator of transcription 4 (STAT4)* and the *protein tyrosine phosphatase N22 (PTPN22)* gene single nucleotide polymorphisms (SNPs), gene–gene interactions and haplotype on type-1 Autoimmune Hepatitis (AIH) risk.

**Results:**

Logistic regression analysis showed that type 1 AIH was significantly higher in carriers of T allele of rs7574865 than those with GG genotype (*P*- value less than 0.001), higher in carriers of C allele of rs7582694 than those with GG genotype (*P*- value < 0.001), and lower in carriers of T allele of rs2476601 than those with CC genotype (*P*- value < 0.001). GMDR model indicated a significant two-locus model (*p* = 0.0100) involving rs7582694 and rs2476601. Participants with GC or CC of rs7582694 and CC of rs2476601 genotype have the highest type 1 AIH risk (*P*- value < 0.001), after covariates adjustment. Haplotype containing the rs7582694-C and rs7574865-T alleles were associated with a statistically increased type 1 AIH risk (*P* < 0.001).

**Materials and Methods:**

Logistic regression was performed to investigate association between SNPs within *STAT4* and *PTPN22* gene and susceptibility to type 1 AIH. Generalized multifactor dimensionality reduction (GMDR) was used to screen the best interaction combinations among the 4 SNPs.

**Conclusions:**

We conclude that rs7574865 and rs7582694 in *STAT4* gene minor alleles, interaction between rs7582694 and rs2476601, and haplotype containing the rs7582694-C and rs7574865-T alleles are associated with increased type 1 AIH risk, but rs2476601 in *PTPN22* gene minor allele is associated with decreased type 1 AIH risk.

## INTRODUCTION

Autoimmune hepatitis (AIH) was a kind of chronic inflammation of the liver, hypergammaglobulinemia and autoantibodies production [1]. A recent study in pediatric patients reported an incidence of 0.4 case per 100 000 children [2]. Another study from Poland reported an incidence of 3 to 4 per 100 000 children [3]. This disease displays female predominance and is considered rare in childhood, although it may occur in very young children [4]. The pathogenesis of AIH was not well known, but could be influenced by both genetic and environmental factors [5]. Mutations in the human leukocyte antigen (HLA) region have been reported associations with some types of autoimmune diseases, including AIH [6, 7].

Signal transducer and activator of transcription 4 (*STAT4*) gene has important role in dendritic cells and macrophages, activated peripheral blood monocytes [8]. The association between *STAT4* and some autoimmune diseases, including rheumatoid arthritis (RA) or systemic lupus erythematosus (SLE) has been reported in several previous studies [9, 10], and in a mouse model, *STAT4* is considered important of Th1-dependent liver injury [11]. A recent study suggested that *STAT4* polymorphism was positively associated with type-1 AIH. rs2476601 in *PTPN22* was a missense single nucleotide polymorphism (SNP), which has been more studied previously [12], and has been reported associations with some types of autoimmune diseases, including RA and SLE [12–15]. A Japanese population base study [16] indicated that *PTPN22* SNPs play an important role in the genetic resistance to autoimmune liver disease. In consideration of the limited number of study on association between *STAT4* and *PTPN22* gene and AIH risk, particularly in Children. In this study, we aimed to investigate the impact of *STAT4* and *PTPN22* gene SNPs, additional gene–gene interaction and haplotype combination on type 1 AIH risk based on Chinese Han population.

## RESULTS

A total of 542 participants (98 boys, 444 girls) consist of 180 AIH patients and 362 normal participants were included in this study. The mean age of all participants is 8.1 ± 3.9 years. The clinical characteristics for cases and controls were shown in Table 1. The distribution of gender and the mean age were not significantly different between cases and controls. The mean of total Bilirubin was 3.87 ± 4.14 mg/ml, albumin was 3.89 ± 0.75 g/L, the rate of SMA and cirrhosis at entry were 56.7% and 30.6 respectively.

**Table 1:** General characteristics of 542 study participants in case and control group

Variables	Case group (*n* = 180)	Normal group (*n* = 362)	*p*-values
Age (year)	7.8 ± 4.1	8.3 ± 3.7	0.154
Girls, *N* (%)	146 (81.11)	298 (82.3)	0.730
Total Bilirubin (mg/ml)	3.87 ± 4.14		
Albumin (3.5–5.0 g/L)	3.89 ± 0.75		
IgG (870–1700 mg/dl)	3085 (2407–3875)		
IAIHG score	16.4 ± 2.3		
SMA, *n* (%)	102 (56.7)		
ANA + (≥ 1:40), *N* (%)	151 (83.9)		
Baseline laboratory values			
AST (< 40 IU/L)	432.4 ± 444.1		
ALT (< 40 IU/L)	484.3 ± 490.5		
ALP (< 112 IU/L)	463.7 ± 210.3		

Note: Means ± standard deviation for age, total Bilirubin, albumin and IAIHG score; Median (inter-quartile range) for IgG.

All genotypes are distributed according to HWE in controls (all *p* values are more than 0.05). The frequencies for rs7574865- T allele and rs7582694- C allele were significantly higher in type 1 AIH cases than control group (30.6% *vs*19.3%, 32.5% *vs*20.0%), and the frequencies for rs2476601- T allele was significantly lower in in type 1 AIH cases than control group (19.7% *vs*30.0%). Logistic regression analysis showed that type 1 AIH was significantly higher in carriers of T allele of rs7574865 than those with GG genotype (GT + TT versus GG), adjusted OR (95% CI) = 1.63 (1.28–.98), and higher in carriers of C allele of rs7582694 than those with GG genotype (GC + CC versus GG), adjusted OR (95% CI) = 1.73 (1.38–2.19). In addition, we also found type 1 AIH risk was significantly lower in carriers of T allele of rs2476601 than those with CC genotype (CT + TT versus CC), adjusted OR (95% CI) = 0.65 (0.44–0.93). However, we did not find any significant association between rs2488457 and type 1 AIH risk after covariates adjustment. (Table 2).

**Table 2:** Genotype and allele frequencies of 4 SNPs between case and control group

Gene/ SNP	Genotypes and Alleles	Frequencies N (%)		OR (95% CI)*	*P*- values	*P*- values for HWE test in controls
		Case (*n* = 180)	Control (*n* = 362)			
STAT4 gene						
rs7574865	Co- dominant					
	GG	89 (49.4)	237 (65.5)	1.00		0.622
	GT	72 (40.0)	110 (30.4)	1.56 (1.24-1.87)	< 0.001	
	TT	19 (10.6)	15 (4.1)	2.13 (1.46-2.97)	< 0.001	
	Dominant					
	GG	89 (49.4)	237 (65.5)	1.00		
	GT + TT	180 (50.6)	125 (34.5)	1.63 (1.28–1.98)	< 0.001	
	Allele, T (%)	110 (30.6)	140 (19.3)			
rs7582694						
	Co- dominant					
	GG	84 (46.7)	232 (64.1)	1.00		0.875
	GC	75 (41.7)	115 (31.8)	1.64 (1.31–2.04)	< 0.001	
	CC	21 (11.7)	15 (4.1)	2.21 (1.68–2.92)	< 0.001	
	Dominant					
	GG	84 (46.7)	232 (64.1)	1.00		
	GC + C	96 (53.3)	130 (35.9)	1.73 (1.38–2.19)	< 0.001	
	Allele, C (%)	117 (32.5)	145 (20.0)			
PTPN22 gene						
rs2488457	Co- dominant					
	GG	105 (58.4)	192 (53.0)	1.00		0.958
	GC	65 (36.1)	143 (39.5)	0.85 (0.57–1.19)	0.528	
	CC	10 (5.6)	27 (7.5)	0.77 (0.52–1.23)	0.476	
	Dominant					
	GG	105 (58.4)	192 (53.0)	1.00		
	GC + C	75 (41.7)	170 (47.0)	0.83 (0.44–1.20)	0.507	
	Allele, C (%)	85 (23.6)	197 (27.2)			
rs2476601	Co- dominant					
	CC	118 (65.6)	184 (50.8)	1.00		0.105
	CT	53 (29.4)	139 (38.4)	0.68 (0.47–0.93)	0.002	
	TT	9 (5.0)	39 (10.8)	0.56 (0.26–0.91)	< 0.001	
	Dominant					
	CC	118 (65.6)	184 (50.8)			
	CT+TT	62 (34.4)	178 (49.2)	0.65 (0.44–0.93)	< 0.001	
	Allele, T (%)	71 (19.7)	217 (30.0)			

*Adjusted for gender and age. Bonferroni correction threshold: *Pc* < 0.0083.

**Figure 1:**
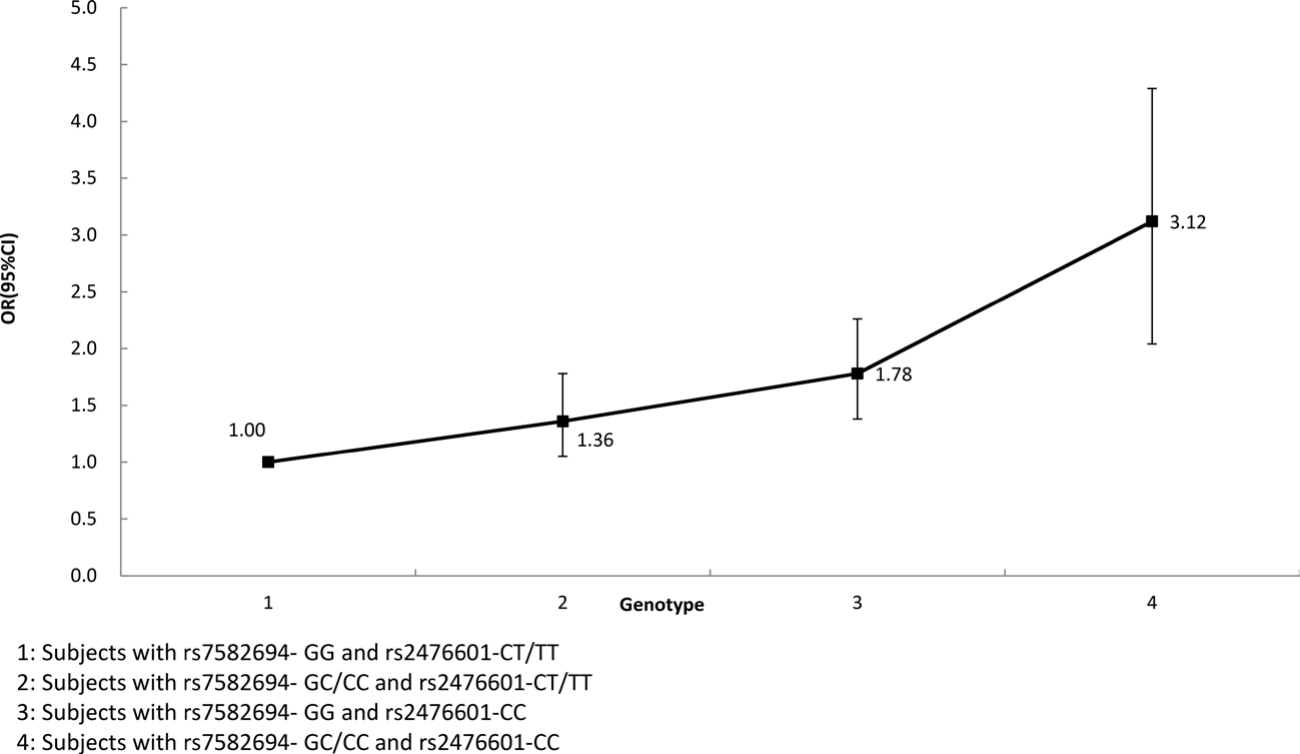
Interaction analysis for rs7582694 and rs2476601 by using logistic regression

GMDR were used to screen the best interaction combinations among 4 SNPs within STAT4 and PTPN22 gene on type 1 AIH risk (Table 3). We found that there is a significant two-locus model (*p* = 0.0100) involving rs7582694 and rs2476601. Overall, the cross-validation consistency of this two- locus model was 10/10, and the testing accuracy was 60.72%. We also conducted stratified analysis for interaction between rs7582694 and rs2476601 by using logistic regression. We found that participants with GC or CC of rs7582694 and CC of rs2476601 genotype have the highest type 1 AIH risk, compared to participants with GG of rs7582694 and CT or TT of rs2476601 genotype, OR (95% CI) = 3.12 (2.04–4.29), after covariates adjustment (Figure 1).

**Table 3:** GMDR investigation on gene–gene interactions within STAT4 and PTPN22 gene

Locus no.	Best combination	Cross-validation consistency	Testing accuracy	p-values^*^
2	rs7582694 rs2476601	10/10	0.6072	0.0100
3	rs7582694 rs2476601 rs7574865	8/10	0.5399	0.1719
4	rs7582694 rs2476601 rs7574865 rs2488457	7/10	0.4958	0.3770

*Adjusted for gender and age.

Pairwise LD analysis was performed between SNPs within the same gene, and D′ value between rs7582694 and rs7574865 was 0.831, D′ value between rs2488457 and rs2476601was 0.761. The most common haplotype in STAT4 gene was rs7582694-G and rs7574865-G haplotype, the frequencies of which were 0.4732 and 0.5547 in case group and control group, and the most common haplotype in PTPN22 gene was rs2488457- G and rs2476601-C haplotype, the frequency of which was 0.47893 and 0.4411 in case group and control group. Haplotype containing the rs7582694-C and rs7574865-T alleles were associated with a statistically increased type 1 AIH risk, OR (95% CI) = 1.85 (1.36–2.47), (*P* < 0.001) (Table 5). But we did not find any haplotype combination in PTPN22 gene associated with type 1 AIH risk (Table 4).

**Table 4:** Haplotype analysis on association between STAT4 gene and type 1 AIH risk

**Haplotypes**	**rs7582694**	**rs7574865**	**Frequencies**		**OR (95%CI)**	*p*-values*
			**Case group**	**Control group**		
H1	G	G	0.4732	0.5547	1.00	—
H2	C	G	0.2236	0.2128	1.14 (0.82–1.64)	0.592
H3	G	T	0.1935	0.1853	1.28 (0.91–1.76)	0.602
H4	C	T	0.1097	0.0472	1.85 (1.36–2.47)	< 0.001

*Adjusted for gender and age. Bonferroni correction threshold: *Pc* < 0.0125.

**Table 5:** Description and Primer sequences for 4 SNPs used for PCR analysis

SNP ID	Chromosome	Functional Consequence	Major/ minor	Restriction enzyme	Primers sequence
STAT4 gene					
rs7574865	2:191099907	Intron variant	G/T	*HpaI*	F: 5′-AAAGAAGTGGGATAAAAAGAAGTTTG-3′ R: 5′-CCACTGAAATAAGATAACCACTGT-3′
rs7582694	2:191105394	Intron variant	G/C	*HpyCH4III*	F: 5′-ATCCAACTCTTCTCAGCCCTT-3′ R: 5′-TCATAATCAGGAGAGAGGAGT-3′
PTPN22 gene					
rs2488457	1:113872746	Intron variant, upstream variant 2KB	G/C	*SacI*	F: 5′-CCATTGAGAGGTTATGCGAGCT-3′ R: 5′-CGCCACCTTGCTGACAACAT-3′
rs2476601	1:113834946	Intron variant, missense	C/T	Xcm*I*	F: 5′-CCAGCTTCCTCAACCACAATAAATG-3′ R: 5′-CAACTGCTCCAAGGATAGATGATGA-3′

## DISCUSSION

In this study, we detected the impact of *STAT4* and *PTPN22* gene polymorphisms on type 1 AIH risk in Chinese Han children; we found that rs7574865 and rs7582694 in *STAT4* gene minor alleles are associated with increased type 1 AIH risk, but rs2476601 in *PTPN22* gene minor allele is associated with decreased type 1 AIH risk. However, we found that *PTPN22-* rs2488457 was not significantly associated with type 1 AIH risk after covariates adjustment. Although several studies [9, 10, 17] have reported the relationship between *STAT4* and *PTPN22* gene SNPs and some others autoimmune diseases, to date, less study focused on relation of *STAT4* and *PTPN22* gene polymorphisms and type 1 AIH risk previously, particularly for Chinese children. The current study was the second study to investigating the association between STAT4 gene polymorphisms and susceptibility to type-1 AIH. Previously Migita et al. [17] suggested that STAT4 polymorphism was positively associated type-1 AIH risk. STAT4 was one type of critical transcription factor involved in the Th1/Th2 cytokine balance regulation [18]. The association between *STAT4* and some autoimmune diseases, including rheumatoid arthritis (RA) or systemic lupus erythematosus (SLE) has been reported in several previous studies [9, 10]. Taylor et al. [19] concluded that STAT4 polymorphism played an important role on susceptibility to SLE. Another two Japanese studies [20, 21] also suggested that STAT4 is a common autoimmune diseases related genetic risk factor, including RA and SLE. A recent meta- analysis [22, 23] demonstrated a significant association between rs7574865- T allele within *STAT4* gene and susceptibility to SLE, RA, T1D and so on. AIH pathogenesis were complex, study indicated that *STAT4* represents a transcription factor, which involved in Th1 and Th17 differentiation [24]. *STAT4* was an important genetic factor for IL-22 production, which plays a pathological role in IL-17-dependent hepatitis [25].

In terms of *PTPN22*, although previous study has showed no significant association between *PTPN22* and AIH [26], to date, just one study [16] have been performed on relationship with type 1 AIH risk, this study suggested that *PTPN22* gene SNP play a protective role on AIH risk. rs2476601 in *PTPN22* was a missense SNP, which has been known as a factor associated with several autoimmune diseases, including RA and SLE [12–14], but this functional SNP was not associated with these autoimmune diseases in other studies [27–29].

In this study, type 1 AIH risk was influenced by both *STAT4* and *PTPN22* gene, so it is interesting to investigate the impact of gene–gene interaction between the two genes on type 1 AIH risk. We found a significant gene–gene interaction between rs7582694 and rs2476601, participants with GC or CC of rs7582694 and CC of rs2476601 genotype have the highest type 1 AIH risk, compared to participants with GG of rs7582694 and CT or TT of rs2476601 genotype. To our knowledge this is the first study for investigating impact of interaction between *STAT4* and *PTPN22* gene on type 1 AIH risk in Chinese population. The underlying mechanisms for this interaction may due to that both SNP were associated with AIH or other autoimmune diseases. We also conducted haplotype analysis in *STAT4* and *PTPN22* gene respectively. We found that haplotype containing the rs7582694-C and rs7574865-T alleles within *STAT4* gene were associated with a statistically increased type 1 AIH risk. But we did not find any haplotype combination within *PTPN22* gene associated with type 1 AIH risk.

The current study also has some limitations. Firstly, limited number of SNP in *STAT4* and *PTPN22* gene are included in current study, and in the future, more SNPs should be included in analysis. Secondly, some environment risk factors should be included in the gene–environment interaction analysis. In addition, the selection bias existed in the participant inclusion and exclusion, so the frequency for the T allele of rs2476601 was higher than that in HapMap data. Thirdly, the results obtained in current study should be checked in other populations, for example, the gender and race difference of this relationship. Lastly, we do not resolve the question of STAT4 SNPs and AIH, Which SNP is dominant or is this purely a haplotype association because of strong LD.

In conclusion, the results of current study indicated that rs7574865 and rs7582694 in *STAT4* gene minor alleles, interaction between rs7582694 and rs2476601, and haplotype containing the rs7582694-C and rs7574865-T alleles are associated with increased type 1 AIH risk, but rs2476601 in *PTPN22* gene minor allele is associated with decreased type 1 AIH risk.

## MATERIALS AND METHODS

### Study population

**Figure 2:**
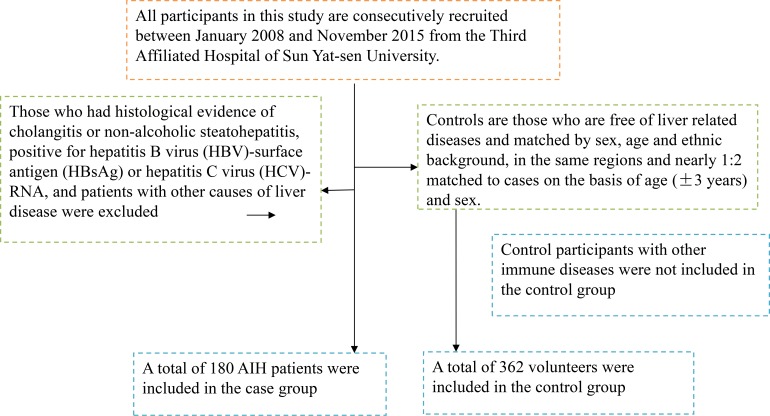
A flowchart on study population selection and exclusion

All participants in this study are consecutively recruited between January 2008 and November 2015 from the Third Affiliated Hospital of Sun Yat-sen University. All AIH patients had been diagnosed according to the scoring system of the International Autoimmune Hepatitis Group [30] and were classified as having type 1 AIH based on antibody profiles. Those patients with clinical evidence of cholangitis or non-alcoholic steatohepatitis, positive for hepatitis B virus (HBV)-surface antigen (HBsAg) or hepatitis C virus (HCV)-RNA, and with other causes of liver disease were excluded from the study, controls are those who are free of liver related diseases and matched by sex, age and ethnic background in the same regions and nearly 1:2 matched to cases on the basis of age (± 3 years), and control participants with other immune diseases were excluded. At last, a total of 542 participants (98 boys, 444 girls) consist of 180 AIH patients and 362 normal participants were included in this study (Figure 2). The mean age of all participants is 8.1 ± 3.9 years. The racial background of all individuals was Chinese Han. Data on demographic information, lifestyle and history of disease for all participants were obtained using a questionnaire administered by trained staffs, including data included coexisting autoimmune diseases, serum levels of alkaline phosphatase and bilirubin and serum levels of ALT, AST and so on. Both anti-nuclear antibodies (ANA) and anti-smooth muscle antibodies (ASMA) were measured by indirect immunofluorescence on HEp-2 cells and cut-off titers for positivity were 1:40. Written informed consent was obtained from all participants. The protocol of this study was approved by the Ethics Committee of the Third Affiliated Hospital of Sun Yat-sen University.

### Genomic DNA extraction and genotyping

We selected SNPs within the *STAT4* and *PTPN22* gene according to the following methods, including: 1) which have been reported associations with autoimmune diseases or risk factors of AIH; 2) minor allele frequency (MAF) greater than 5%. Taking into account the limitations of human, material and financial resources, a total of two SNPs of *STAT4* gene and two SNPs of *PTPN22* gene were selected for genotyping in the study: rs7582694 and rs7574865 within STAT4, rs2476601 and rs2488457 within *PTPN22*. Genomic DNA is extracted from EDTA-treated whole blood, using the DNA Blood Mini Kit (Qiagen, Hilden, Germany) according to the manufacturer’s instructions. The four SNPs were determined by the polymerase chain reaction–restriction fragment length polymorphism (PCR–RFLP) method [31]. PCR primer sequences for each polymorphism are shown in Table 5. The PCR reactions were carried out in a final volume of 20 μl containing: 10 × PCR buffer, 4.5 mMMgCl_2_ (Roche, Germany), 0.4 mM of each dNTP (Fermentas, Germany), 10 pmol of each primer, 32 ng template DNA, 1 U Taq DNA polymerase (Roche, Germany) and sterile distilled water up to 20 μl.

For the two SNPs within *STAT4* gene*,* rs7574865 was a 147-bp PCR product and was digested with restriction enzyme and electrophoresed on a 2.5% polyacrylamide gel. Rs7574865 was a 338-bp PCR product was digested with restriction enzyme and electrophoresed on a 3.0% polyacrylamide gel. For the two SNPs within *PTPN22* gene*,* the PCR products were incubated with restriction enzymes for 1 or 16 hours. 4% agarose gel with Gold View (SBS Genentech, Beijing, China) was used to visualize the obtained digestion products. 20% of PCR-amplified DNA samples were examined by direct sequencing to confirm the genotyping results, which was 100% concordant. Amplification conditions started with an initial denaturation step of 6 min at 94°C, followed by 35 cycles of 40 s denaturation (94°C), 30 s annealing (56°C) and 40 s extension (72°C), ended by a final extension for 5 min (72°C).

### Statistical analysis

The means and standard deviations (SDs) were calculated for normally distributed continuous variables and were analyzed using Student’s *t* test or one-way analysis of variance. Percentages were calculated for categorical variables and were analyzed using χ^2^ test. Departure from Hardy-Weinberg equilibrium (HWE) in cases and controls was tested using Pearson χ^2^ goodness-of-fit test. Haplotype analysis and Pairwise LD analysis were investigated by using SNPStats (available online at http://bioinfo.iconcologia.net/SNPstats). Logistic regression was performed to investigate association between SNPs within *STAT4* and *PTPN22* gene and susceptibility to type 1 AIH. Bonferroni correction was applied in case of multiple comparisons using the formula pc = p/n (pc represents corrected value where n is the number of comparisons made). Generalized multifactor dimensionality reduction (GMDR) [32] was used to screen the best interaction combinations among the 4 SNPs, some parameters such as cross-validation consistency, the testing balanced accuracy, and the sign test were calculated. Permutation testing is also conducted to gain empirical *P* values of prediction accuracy as a benchmark based on 10,000 shuffles. A sign test or a permutation test (providing empirical *p*-values) for prediction accuracy can be used to measure the significance of an identified model.

## References

[1] Czaja AJ, Manns MP (2010). Advances in the diagnosis, pathogenesis, and management of autoimmune hepatitis. Gastroenterology.

[2] Deneau M, Jensen MK, Holmen J, Williams MS, Book LS, Guthery SL (2013). Primary sclerosing cholangitis, autoimmune hepatitis, and overlap in Utah children: epidemiology and natural history. Hepatology.

[3] Woynarowski M, Wozniak M, Pawlowska M, Lebensztejn D,Socha J, ChlebcewiczSzuba W, Chmurska-Motyka T, Czaja-Bulsa G, Gorczyca A, Iwanczak B, Korczowski B, Kuydowicz G, Liberek A (2008). Autoimmune hepatitis in Polish children: healthcare facilities, epidemiology, and standards of care assessed by a pediatric autoimmune hepatitis group. Exp Clin Hepatol.

[4] Gregorio GV, Portmann B, Reid F, Donaldson PT, Doherty DG, McCartney M, Mowat AP, Vergani D, Mieli-Vergani G (1997). Autoimmune hepatitis in childhood: a 20-year experience. Hepatology.

[5] Longhi MS, Ma Y, Mieli-Vergani G, Vergani D (2010). Aetiopathogenesis of autoimmune hepatitis. J Autoimmun.

[6] Seki T, Ota M, Furuta S, Fukushima H, Kondo T, Hino K, Mizuki N, Ando A, Tsuji K, Inoko H, Kiyosawa K (1992). HLA class II molecules and autoimmune hepatitis susceptibility in Japanese patients. Gastroenterology.

[7] Umemura T, Katsuyama Y, Yoshizawa K, Kimura T, Joshita S, Komatsu M, Matsumoto A, Tanaka E, Ota M (2014). Human leukocyte antigen class II haplotypes affect clinical characteristics and progression of type 1 autoimmune hepatitis in Japan. PLoS One.

[8] Kaplan MH (2005). STAT4: a critical regulator of inflammation in vivo. Immunol Res.

[9] Remmers EF, Plenge RM, Lee AT, Graham RR, Hom G, Behrens TW, de Bakker PI, Le JM, Lee HS, Batliwalla F (2007). STAT4 and the risk of rheumatoid arthritis and systemic lupus erythematosus. N Engl J Med.

[10] Sugiura T, Kawaguchi Y, Goto K, Hayashi Y, Tsuburaya R, Furuya T, Gono T, Nishino I, Yamanaka H (2012). Positive association between STAT4 polymorphisms and polymyositis/ derma- tomyositis in a Japanese population. Ann Rheum Dis.

[11] Gao B (2005). Cytokines, STATs and liver disease. Cell Mol Immunol.

[12] Begovich AB, Carlton VE, Honigberg LA, Schrodi SJ, Chokkalingam AP, Alexander HC, Ardlie KG, Huang Q, Smith AM, Spoerke JM, Conn MT, Chang M, Chang SY (2004). A missense single-nucleotide polymorphism in a gene encoding a protein tyrosine phosphatase (PTPN22) is associated with rheumatoid arthritis. Am J Hum Genet.

[13] Hinks A, Barton A, John S, Bruce I, Hawkins C, Griffiths CE, Donn R, Thomson W, Silman A, Worthington J (2005). Association between the PTPN22 gene and rheumatoid arthritis and juvenile idiopathic arthritis in a UK population: further support that PTPN22 is an autoimmunity gene. Arthritis Rheum.

[14] Orozco G, Sánchez E, González-Gay MA, López-Nevot MA, Torres B, Cáliz R, Ortego-Centeno N, Jiménez-Alonso J (2005). Association of a functional single-nucleotide polymorphism of PTPN22, encoding lymphoid protein phosphatase, with rheumatoid arthritis and systemic lupus erythematosus. Arthritis Rheum.

[15] Orrú V, Tsai SJ, Rueda B, Fiorillo E, Stanford SM, Dasgupta J, Hartiala J, Zhao L, Ortego-Centeno N, D’Alfonso S, Arnett FC, Wu H, Italian Collaborative Group (2009). A loss-of-function variant of PTPN22 is associated with reduced risk of systemic lupus erythematosus. Hum Mol Genet.

[16] Umemura T, Joshita S, Yamazaki T, Komatsu M, Katsuyama Y, Yoshizawa K, Tanaka E, Ota M (2016). Genetic Association of PTPN22 Polymorphisms with Autoimmune Hepatitis and Primary Biliary Cholangitis in Japan. Sci Rep.

[17] Alvarez F, Berg PA, Bianchi FB, Bianchi L, Burroughs AK, Cancado EL, Chapman RW, Cooksley WG, Czaja AJ (1999). International Autoimmune Hepatitis Group Report: review of criteria for diagnosis of autoimmune hepatitis. J Hepatol.

[18] Piotrowski P, Lianeri M, Wudarski M, Olesinska M, Jagodziński PP (2012). Contribution of STAT4 gene single-nucleotide polymorphism to systemic lupus erythematosus in the Polish population. Mol Biol Rep.

[19] Lou XY, Chen GB, Yan L, Ma JZ, Zhu J, Elston RC, Li MD (2007). A generalized combinatorial approach for detecting gene-by gene and gene-by- environment interactions with application to nicotine dependence. Am J Hum Genet.

[20] Kobayashi S, Ikari K, Kaneko H, Kochi Y, Yamamoto K, Shimane K, Nakamura Y, Toyama Y, Mochizuki T, Tsukahara S (2008). Association of STAT4 with susceptibility to rheumatoid arthritis and systemic lupus erythematosus in the Japanese population. Arthritis Rheum.

[21] Namjou B, Sestak AL, Armstrong DL, Zidovetzki R, Kelly JA, Jacob N, Ciobanu V, Kaufman KM, Ojwang JO, Ziegler J, Quismorio FP, Reiff A, Myones BL (2009). High- density genotyping of STAT4 reveals multiple haplotypic associations with systemic lupus erythematosus in different racial groups. Arthritis Rheum.

[22] Liang YL, Wu H, Shen X, Li PQ, Yang XQ, Liang L, Tian WH, Zhang LF, Xie XD (2012). Association of STAT4 rs7574865 polymorphism with autoimmune diseases: a meta-analysis. Mol Biol Rep.

[23] Tong G, Zhang X, Tong W, Liu Y (2013). Association between polymorphism in STAT4 gene and risk of rheumatoid arthritis: a meta-analysis. Hum Immunol.

[24] Murphy KM, Ouyang W, Szabo SJ, Jacobson NG, Guler ML, Gorham JD, Gubler U, Murphy TL (1999). T helper differentiation proceeds through Stat1-dependent, Stat4-dependent and Stat4-independent phases. Curr Top Microbiol Immunol.

[25] Xu M, Morishima N, Mizoguchi I, Chiba Y, Fujita K, Kuroda M, Iwakura Y, Cua DJ, Yasutomo K, Mizuguchi J, Yoshimoto T (2011). Regulation of the development of acute hepatitis by IL-23 through IL-22 and IL-17 production. Eur J Immunol.

[26] de Boer YS, van Gerven NM, Zwiers A, Verwer BJ, van Hoek B, van Erpecum KJ, Beuers U, van Buuren HR, Drenth JP, den Ouden JW, Verdonk RC, Koek GH, Brouwer JT (2014). Dutch Autoimmune Hepatitis Study Group; LifeLines Cohort Study; Study of Health in Pomerania. Genome-wide association study identifies variants associated with autoimmune hepatitis type 1. Gastroenterology.

[27] Ban Y, Tozaki T, Taniyama M, Tomita M, Ban Y (2005). The codon 620 single nucleotide polymorphism of the protein tyrosine phosphatase-22 gene does not contribute to autoimmune thyroid disease susceptibility in the Japanese. Thyroid.

[28] Ikari K, Momohara S, Inoue E, Tomatsu T, Hara M, Yamanaka H, Kamatani N (2006). Haplotype analysis revealed no association between the PTPN22 gene and RA in a Japanese population. Rheumatology (Oxford).

[29] Kawasaki E, Awata T, Ikegami H, Kobayashi T, Maruyama T, Nakanishi K, Shimada A, Uga M, Kurihara S, Kawabata Y, Tanaka S, Kanazawa Y, Lee I (2006). Systematic search for single nucleotide polymorphisms in a lymphoid tyrosine phosphatase gene (PTPN22): association between a promoter polymorphism and type 1 diabetes in Asian populations. Am J Med Genet A.

[30] Migita K, Nakamura M, Abiru S, Jiuchi Y, Nagaoka S, Komori A, Hashimoto S, Bekki S, Yamasaki K, Komatsu T, Shimada M, Kouno H, Hijioka T (2013). Association of STAT4 Polymorphisms with Susceptibility to Type-1 Autoimmune Hepatitis in the Japanese Population. PLoS One.

[31] Watford WT, Hissong BD, Bream JH, Kanno Y, Muul L, O’Shea JJ (2004). Signaling by IL-12 and IL-23 and the immunoregulatory roles of STAT4. Immunol Rev.

[32] Taylor KE, Remmers EF, Lee AT, Ortmann WA, Plenge RM, Tian C, Chung SA, Nititham J, Hom G, Kao AH, Demirci FY, Kamboh MI, Petri M (2008). Specificity of the STAT4 genetic association for severe disease manifestations of systemic lupus erythematosus. PLoS Genet.

